# Granulocyte macrophage‐colony stimulating factor and keratinocyte growth factor control of early stages of differentiation of oral epithelium

**DOI:** 10.1111/eos.12867

**Published:** 2022-04-22

**Authors:** Ridhima Das, Maria Justina Roxana Virlan, Victoria Xenaki, Keerthi K. Kulasekara, Ochiba Lukandu, Evelyn Neppelberg, Olav K. Vintermyr, Anne. Chr. Johannessen, Bogdan Calenic, Daniela Elena Costea

**Affiliations:** ^1^ Center for Cancer Biomarkers CCBIO and Gade Laboratory for Pathology Department of Clinical Medicine Faculty of Medicine University of Bergen Bergen Norway; ^2^ Department of Oral Rehabilitation and Department of Biochemistry ‘Carol Davila’ University of Medicine and Pharmacy Bucharest Romania; ^3^ Department of Clinical Dentistry Faculty of Medicine University of Bergen Bergen Norway; ^4^ Department of Pharmacy & Applied Science College of Science, Health & Engineering La Trobe University Bendigo Victoria Australia; ^5^ Maxillofacial Surgery and Pathology, School of Dentistry Moi University Eldoret Kenya; ^6^ Department of Oral and Maxillofacial Surgery Head and Neck Clinic Haukeland University Hospital Bergen Norway; ^7^ Department of Pathology Haukeland University Hospital Bergen Norway

**Keywords:** cell differentiation, cell proliferation, mouth mucosa

## Abstract

Oral epithelial differentiation is known to be directed by underlying fibroblasts, but the responsible factor(s) have not been identified. We aimed here to identify fibroblast‐derived factors responsible for oral epithelial differentiation. Primary normal human oral keratinocytes and fibroblasts were isolated from healthy volunteers after informed consent (*n* = 5) and 3D‐organotypic (3D‐OT) cultures were constructed. Various growth factors were added at a range of 0.1‐100 ng/ml. 3D‐OTs were harvested after ten days and assessed histologically, by immunohistochemistry and the TUNEL method. Epithelium developed in 3D‐OT without fibroblasts showed an undifferentiated phenotype. Addition of granulocyte macrophage‐colony stimulating factor (GM‐CSF) induced expression of cytokeratin 13 in suprabasal cell layers. Admixture of GM‐CSF and keratinocyte growth factor (KGF) induced, in addition, polarization of epidermal growth factor (EGF) receptor and β1‐integrin to basal cell layer and collagen IV deposition. Terminal differentiation with polarization of TUNEL‐positive cells to superficial layers occurred only in the presence of fibroblasts in collagen gels either in direct contact or at distance from normal oral keratinocytes. Taken together, these results show that major aspects of oral epithelial differentiation are regulated by the synergic combination of GM‐CSF and KGF. However, the terminal stage seems to be controlled by other yet unidentified fibroblast‐derived diffusible factor(s).

## INTRODUCTION

It is well established that the molecular interactions between epithelium and mesenchyme is essential for keratinocyte proliferation, differentiation and repair in both skin and oral mucosa [1, 2, 3, 4]. Previous studies on three dimensional organotypic (3D‐OT) in vitro models, including our own, demonstrated that fibroblasts were essential for the resemblance of the tissues reconstructed 3D in vitro with the in vivo human oral mucosa [1, 5, 6]. Several studies have tested various fibroblast‐derived factors for their role on oral epithelium differentiation; keratinocyte growth factor (KGF) was found to stimulate proliferation of oral keratinocytes but not influence their differentiation when added alone to 3D‐OT models constructed with keratinocyte only (3D‐OT monocultures) [1]. This was in contrast to the effect of KGF on dermal keratinocytes in 3D‐OT cultures [7]. The hematopoietic granulocyte macrophage‐colony stimulating factor (GM‐CSF) which is synthesized by macrophages, T cells, mast cells, natural killer cells, endothelial cells, and fibroblasts and which normally functions as a cytokine facilitating development of the immune system and promoting the defense against infections was found to also regulate dermal keratinocyte growth and differentiation [8]. Fibroblast‐keratinocyte co‐cultures in fetal skin models strongly enhanced the expression of GM‐CSF by fetal skin cells [9], while dermal keratinocyte‐released interleukin 1α (Il‐1α) induced the expression of both KGF and GM‐CSF in dermal fibroblasts [10]. These studies indicated GM‐CSF as a growth factor involved in epithelial‐mesenchymal interactions, but its effect on oral epithelial morphogenesis has not been tested so far. Much of the knowledge on epithelial‐mesenchymal interactions comes from studies on skin models, but there are distinctive signals for epithelial differentiation of oral and dermal fibroblasts [11] and there is a gap of knowledge on how fibroblasts regulate the differentiation of oral epithelium. The aim of this study was to identify the fibroblast‐derived factors responsible for oral epithelial differentiation, and for this purpose several growth factors were tested, such as epidermal growth factor (EGF), KGF, GM‐CSF, transforming growth factor α (TGFα), IL‐1α, hepatocyte growth factor (HGF), and stem cell factor (SCF). The present study presents data in support for the control of oral epithelial differentiation by the underlying mesenchyme via soluble factors synthesized by oral fibroblasts. GM‐CSF, alone or in combination with KGF, was able to control several steps of differentiation, except its terminal stages. This indicates that other yet unidentified fibroblast‐derived soluble factor(s) may be responsible for regulation of terminal differentiation in oral epithelia.

## MATERIAL AND METHODS

### Human donors

Eighteen samples of normal human oral mucosa were obtained from healthy donors undergoing wisdom tooth extraction (details in Table 1). Seven samples were snap‐frozen in isopentane and six samples were formalin‐fixed and embedded in paraffin. Cells successfully isolated and propagated from five samples were used for growing of 3D‐OT cultures. The study was approved by the Ethics Committee of the University of Bergen (REK 2010/481) and the samples were collected after informed consent.

**TABLE 1:** Demographics (age, gender, tobacco use) of the donors included in the study and the usage of the tissues harvested

Donor ID	Age	Gender	Tobacco use	Usage
41	44	M	No	Frozen
42	24	M	No	Frozen
49	20	M	No	Frozen
62	20	F	No	Frozen
77	35	F	No	Frozen
92	34	M	No	Frozen
98	23	M	No	Frozen
43	20	F	No	FFPE
44	35	M	No	FFPE
45	31	M	No	FFPE
55	23	M	No	FFPE
57	26	F	No	FFPE
59	22	M	No	FFPE
48	25	F	No	Isolating cells
60	22	M	No	Isolating cells and FFPE
63	24	F	No	Isolating cells
80	24	F	No	Isolating cells
93	25	M	No	Isolating cells

Abbreviation: FFPE, formalin fixed and paraffin embedded.

### Primary cell cultures

Primary human normal oral fibroblasts and keratinocytes were isolated as previously described [1]. Normal oral keratinocytes were routinely grown on plastic surfaces (Nunc) with no feeding layers, in keratinocyte serum free medium supplemented with 1 ng/ml human recombinant EGF (GibcoBRL), 25 µg/ml bovine pituitary extract (GibcoBRL), 2 mM L‐glutamine (GibcoBRL), 100 U/ml penicillin (GibcoBRL), 100 µg/ml streptomycin (GibcoBRL), 0.25 µg/ml amphotericin B (GibcoBRL). Normal oral fibroblasts were grown in Minimum Essential Medium Eagle (Sigma) supplemented with 10% fetal calf serum (Sigma), 2 mM L‐glutamine, 100 U/ml penicillin, 100 µg/ml streptomycin, 0.25 µg/ml amphotericin B. To reduce the variability, one single batch of fetal calf serum has been used throughout the studies.

### 3D‐OT cell culture procedures

**FIGURE 1:**
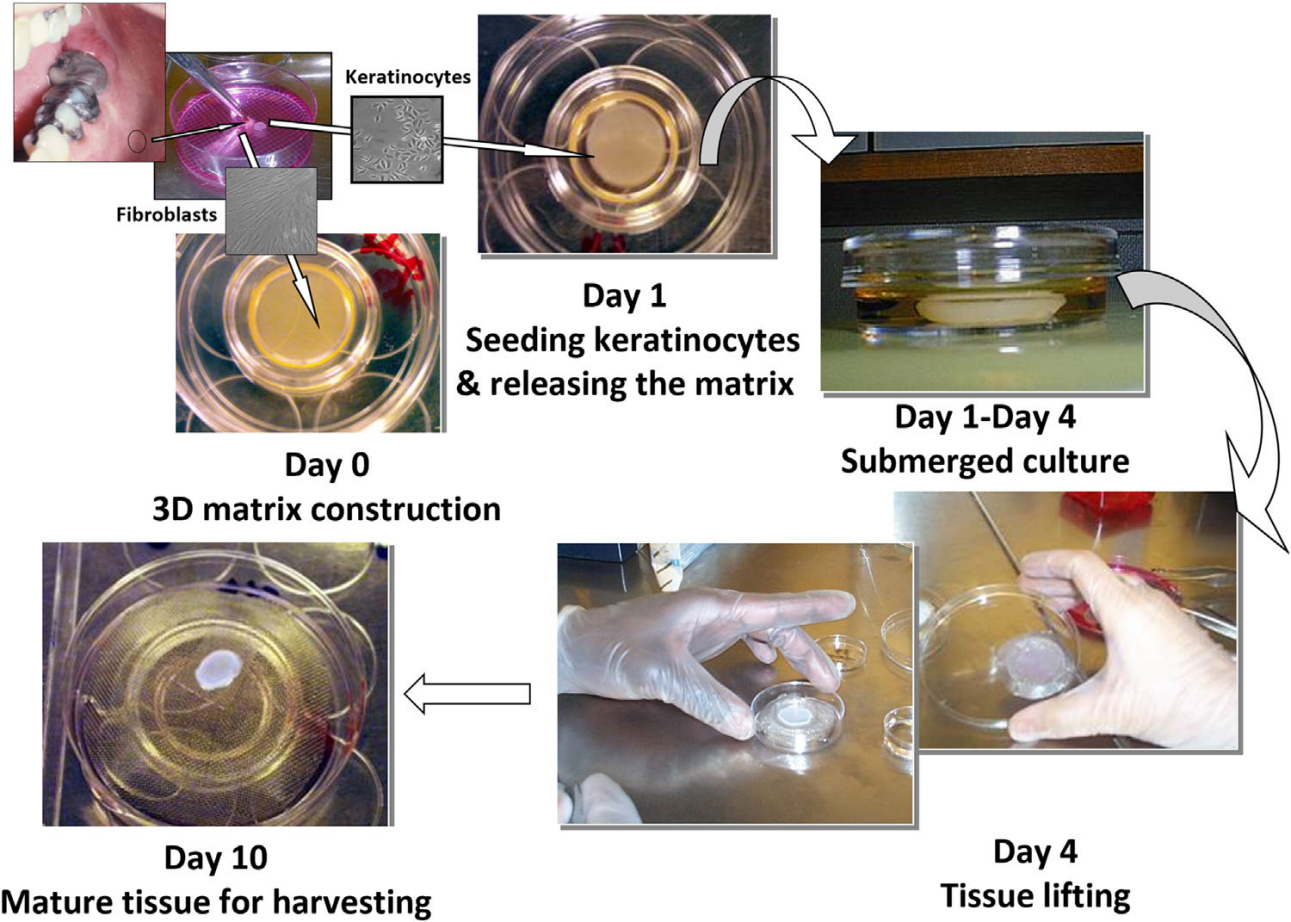
Step‐by‐step laboratory procedures for construction of three dimensional organotypic cultures using primary cells isolated from normal human oral mucosa

Simple collagen gels (700 µl for each culture) were prepared on ice by mixing 7 vol. (3.40 mg/ml) of rat tail collagen type I (Collaborative Biomedical), 2 vol. reconstitution buffer (261 mM NaHCO3, 150 mM NaOH, 200 mM HEPES) pH 8.15, 1 vol. Dulbecco's Modified Eagle Medium (DMEM) 10x (Sigma) and 1 vol. fetal calf serum. Fibroblast‐containing collagen matrices were prepared by mixing 1 vol. fetal calf serum containing 0.5 × 10^6^/ml normal oral fibroblasts in passages 2–4. Seven hundred µl of the prepared matrix was pipetted in 24 well plates and let for 30 min in the incubator to turn to gel. Normal oral fibroblasts growth medium (1 ml/well) was then added over the matrices. After 24 h, the medium on top of the gels was removed and normal oral keratinocytes (0.5 × 10^6^ cells/culture) at second passage were added in 1 ml of their growth medium [1]. After 24 to 48 h, the cultures were lifted on the air‐liquid interface. The flow of procedures for construction of 3D‐OT cultures is presented in Figure 1. The suspended 3D organotypic cultures were grown in serum free medium comprising DMEM and Ham's F‐12 nutrient mix in 3:1, supplemented with 1 µM hydrocortisone, 0.8 µM insulin, 0.25 mM transferrin, 0.25 mM L‐ascorbic acid, 15–30 µM linoleic acid, 15 µM bovine serum albumin, 2 mM L‐glutamine (all from Sigma). Sandwich models were manufactured by interposing a layer of collagen biomatrix (500 µl) between the epithelial compartment and the fibroblast containing matrix. Human growth factors (EGF, KGF, GM‐CSF, TGFα, IL‐1α, HGF, SCF –Sigma) were added to the culture media of some of the collagen simple matrix cultures at a range of 0.1‐100 ng/ml, as summarized in Table 2. All cultures were maintained at 37°C in 5% CO_2_ incubators for the whole duration of the experiment. All cultures were harvested on day 10 of co‐culture. One half of each culture was snap frozen in isopentane pre‐chilled in liquid nitrogen and the other fixed in 4% buffered formalin pH 7.15 and embedded in paraffin. Experiments (run in duplicates) were repeated 5 times, each time with primary cells isolated from different patients (*n* = 5 donors).

**TABLE 2:** Table showing the growth factors added to the 3D‐OT cultures and the outcomes of the different combinations in terms of the presence of different epithelial cell layers: Presence of spinous cell layer indicates that the cells underwent early differentiation in those culture conditions; presence of superficial cell layer indicates that the cells underwent full differentiation in those culture conditions

Growth factor/fibs	Concentration(ng/ml)	Basal cell layer	Spinous cell layer	Superficial cell layer
none	–	Yes	No	No
EGF	10	Yes	No	No
EGF	10			
+ KGF	0.1 1 10 100	Yes Yes Yes Yes	No No No No	No No No No
EGF	10			
+ GM‐CSF	10	Yes	No	No
EGF	10			
+ HGF	10	Yes	No	No
EGF	10			
+TGFα +IL‐1α	10 10	Yes Yes	No No	No No
KGF	0.1 1 10 100	Yes Yes Yes Yes	No No No No	No No No No
KGF	10			
+ GM‐CSF	0.1 1 10 100	Yes Yes Yes Yes	No No Yes Yes	No No No No
KGF	10			
+ HGF	10	Yes	No	No
KGF	10			
+ GM‐CSF	10			
+ TGFα	10	Yes	Yes	No
GM‐CSF	0.1 1 10 100	Yes Yes Yes Yes	No No Yes Yes	No No No No
GM‐CSF	10			
+ HGF	10	Yes	Yes	No
GM‐CSF	10			
+ TGFα	10			
+ HGF	10	Yes	Yes	No
TGFα	10	Yes	No	No
TGFα	10			
+ HGF	10	Yes	No	No
IL‐1α	10	Yes	No	No
HGF	10	Yes	No	No
SCF	10	Yes	No	No
All growth factors	10	Yes	No	No
fibs	–	Yes	Yes	Yes
sandwich	–	Yes	Yes	Yes

Abbreviations: EGF, epidermal growth factor; GM‐CSF, granulocyte macrophage‐colony stimulating factor; HGF, hepatocyte growth factor; KGF, keratinocyte growth factor; SCF, stem cell factor.; TGFα, transforming growth factor α.

### ELISA

Conditioned media was collected from normal oral fibroblasts cells (*n* = 5 donors) maintained in monocellular (normal oral fibroblasts only) 3D cultures at similar passages, and analyzed for levels of various growth factors and cytokines by using the Widescreen Human Cancer Panel 2 (Novagen) with Luminex beads (R&D Systems).

### Immunohistochemical staining

The immunohistochemical staining was carried out using the DAKO autostainer – Universal Staining System (DAKO). Five µm thick fresh or formalin fixed, paraffin embedded sections were used. The staining for E‐cadherin was carried on fresh frozen sections fixed for 30 s in 50% cold acetone, and afterward for 5 min in 100% acetone before washing in distilled water. All sections were processed then as previously reported [1]. The sections were incubated with the primary antibody for 60 min, and afterward with the secondary antibody conjugated with horseradish peroxidase labelled polymer (EnVision+ System; DAKO) for 30 min. Primary antibodies (all IgG1) and titrations used in this study were as follows: Ki‐67, MIB‐1clone, 1:50 (DAKO); cytokeratin 13 (CK13), KS‐1A3 clone, 1:400 (Novocastra Laboratories); β1‐integrin, K20 clone, 1:2000 (DAKO), EGF‐R, E30 clone, 1:100 (DAKO), E‐cadherin, HECD‐1 clone, 1:9000 (R&D Systems), collagen IV, CIV221 clone, 1:25 (DAKO). Presence of antigen was visualised with DAB+ (3,3′‐diaminobenzidine, DAKO). Biopsies of normal human oral mucosa served as reference controls (those marked as frozen and formalin fixed and embedded in paraffin in Table 1). Specimens incubated with antibody diluent (DAKO) or CD 3 antibody (having the same isotype as the antibodies tested in the study) instead of primary antibody were used as negative controls.

### TUNEL method

Cell death was detected by the TUNEL method (terminal deoxynucleotidyl transferase‐mediated dUTP in situ nick end‐labelling) on formalin fixed paraffin embedded sections [12]. For positive controls, specimens were treated with 0.5 mg/ml DNase (Roche Diagnostics) in tris‐buffered saline for 15 min at 37°C prior to incubation with bovine serum albumin. The specificity of the TUNEL reaction was tested by substituting the biotinylated dUTP in the TUNEL labelling mixture with unbiotinylated dUTP (Roche) in excess. TUNEL positive keratinocytes found within the basal cell layer were considered spontaneously apoptotic cells, while TUNEL positive cells found at the superficial cell layer on top of the epithelium were considered terminally differentiated keratinocytes [13].

### Evaluation of samples and statistical analysis

ELISA results are presented with values normalized for 10^6^ cells; data were analyzed using t‐test with a level of significance set at 5% (SPSS 11.0). The data is presented as mean +/‐ SD. Tissue sections (5µm) from paraffin embedded specimens, stained with Haematoxylin‐eosin, were morphometrically analyzed by a computer‐based optical image analyzer (analySIS 11.0 Pro Soft Imaging System). Ki‐67 /proliferation index was determined as the percentage of positive cells among all cells of the basal cell layer per 400µm length of the epithelial‐mesenchymal interface. The measurements and counts were done at 200 fold magnification on a standard microscope (LeikaDMLM) on six consecutive fields situated 200 µm apart. Statistical analysis was performed using Wilcoxon paired test with a level of significance set at 5% (SPSS 11.0).

## RESULTS

### Effects of KGF and GM‐CSF, alone or in combination, on epithelial cell proliferation and thickness of in vitro reconstructed normal human oral epithelium

**FIGURE 2:**
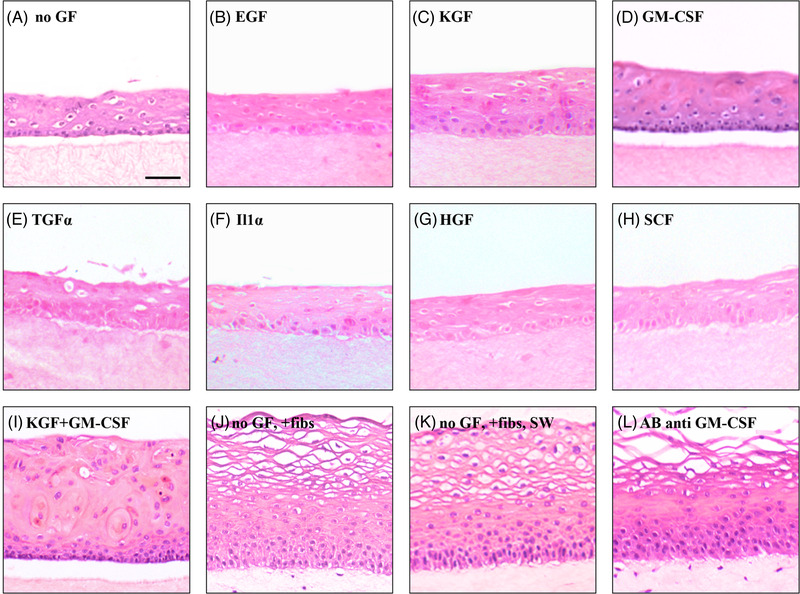
Effects of fibroblast‐derived diffusible factors, granulocyte macrophage‐colony stimulating factor (GM‐CSF) alone or in combination with keratinocyte growth factor (KGF), and an antibody against GM‐CSF on tissue morphology in in vitro reconstituted human oral epithelium. Three dimensional organotypic cultures were constructed with primary normal human oral keratinocytes on top of either simple collagen type I gels (A‐I) and the cultures were grown in medium with various growth factors at 10 ng/ml: epidermal growthfactor (B), KGF (C), GM‐CSF (D), transforming growth factor alpha (E), interleukin 1 alpha (F), hepatocyte growthfactor (G), stem cell factor (H), or a combination of growth factors KGF and GM‐CSF (I). Other three dimensional models were constructed by seeding normal oral keratinocytes on top of human fibroblast‐containing collagen gels (J‐L) either in direct contact (J and L) or at distance through a layer of simple collagen layer (sandwich models ‐ K). An antibody against GM‐CSF (L) was added to three dimensional cultures with fibroblast‐containing collagen type I gels. All cultures were harvested on day 10 of co‐culture. One half of each culture was fixed in 4% buffered formalin pH 7.15 and embedded in paraffin. Sections of representative cultures stained with haematoxylin & eosin are shown. Scale bar = 100 µm

**FIGURE 3:**
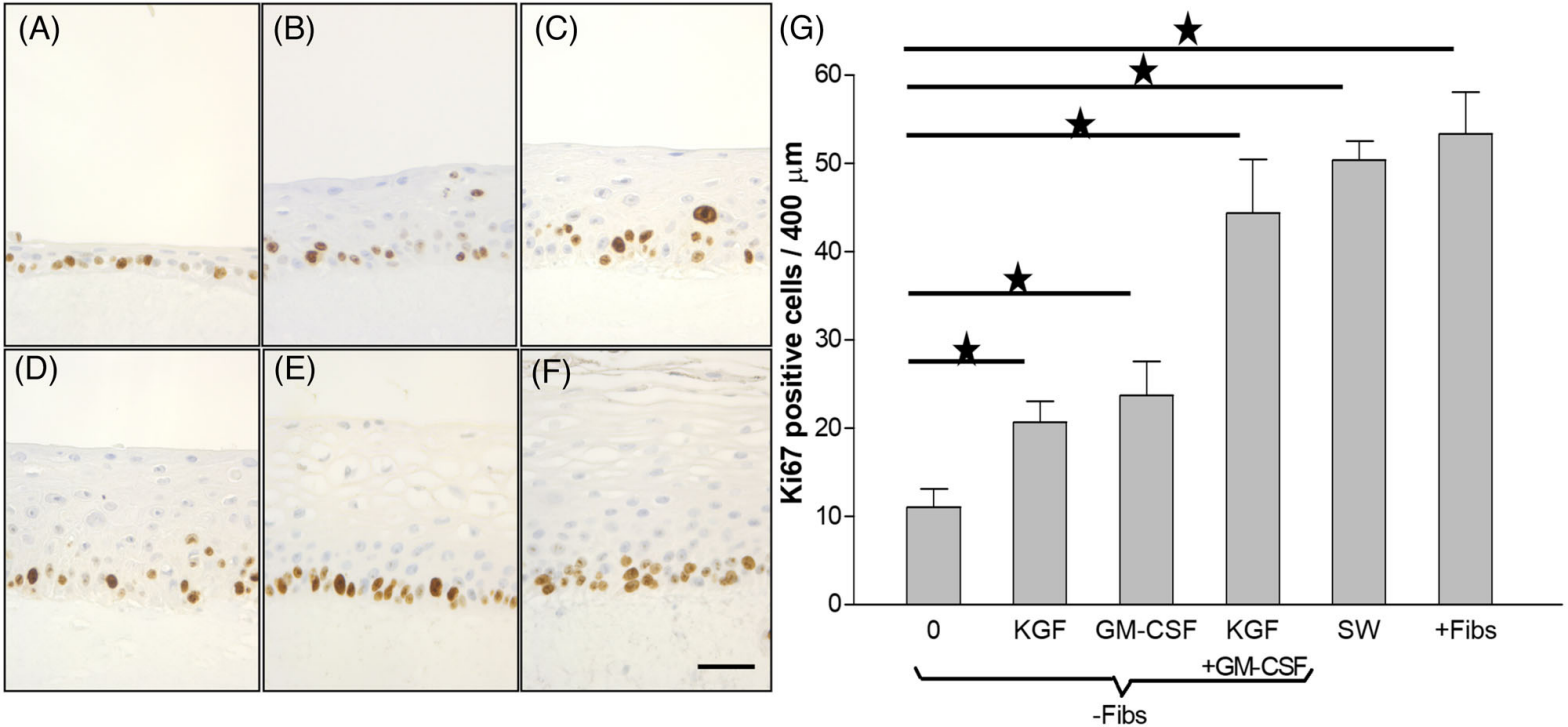
The effect of fibroblast‐derived soluble factors on oral epithelial cell proliferation in in vitro reconstituted human oral epithelium. Human oral epithelium was in vitro reconstituted on simple collagen matrix (‐Fibs) or on collagen gels populated with fibroblasts in direct contact (+Fibs) or at distance from the epithelial compartment in sandwich models and immunohistochemistry for Ki67 was performed in order to detect the proliferating cells. Immunohistochemistry pictures showing normal oral keratinocytes cells from the same patient grown on top of collagen matrices without any additional growth factors (A), with 10 ng/ml keratinocyte growth factor (KGF) (B), with 10 ng/ml granulocyte macrophage‐colony stimulating factor (GM‐CSF) (C), with a combination of 10 ng/ml KGF and 10 ng/ml GM‐CSF (D), in sandwich models (E) and on top of fibroblasts‐populated collagen gels (F). Bars (mean of duplicate three dimensional cultures constructed with cells from *n* = 5 donors) and standard deviations show the percentage of Ki67 positive cells among the cells of the basal cell compartment (G)

**FIGURE 4:**
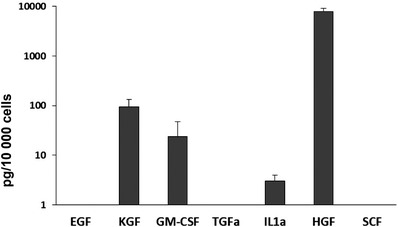
Quantification of growth factors synthesized by normal oral fibroblasts in three dimensional monocellular cultures. Graph showing secretion of various growth factors and cytokines determined by ELISA for normal oral fibroblasts grown in three dimensional biomatrices. Bars (mean of triplicate 3D cultures containing fibroblasts only in collagen gels, constructed with cells from *n* = 5 donors) and standard deviations are shown (*n* = 5)

The oral mucosa formed by growing primary normal human buccal keratinocytes on simple collagen gels in absence of fibroblasts displayed a thin epithelium (Figure 2A) with low cell proliferation (Figure 3). Presence of fibroblasts in the collagen matrix, either in direct contact with keratinocytes (Figure 2J) or at distance (in the ‘sandwich models’‐ Figure 2K) induced an increase in cell proliferation (Figure 3). No differences between cell proliferation indices or the phenotypes of reconstructed oral epithelia could be detected between cultures with direct keratinocyte‐fibroblast contact and cultures with keratinocytes at distance from fibroblasts (Figure 3). Analysis of conditioned medium from 3D gels populated with fibroblasts showed that fibroblasts secreted HGF, KGF, GM‐CSF, and IL‐1α when grown in 3D cultures in vitro (Figure 4). Both KGF and GM‐CSF at concentrations higher than 1 ng/ml, either alone or in combination, increased cell proliferation in the basal cell layer (Figure 3). EGF, TGFα, IL‐1α, HGF, or SCF did not alter epithelial thickness (Figure 2B, E‐H), or epithelial cell proliferation in 3D‐OT monocultures of keratinocytes (Figure 4).

### Effects of GM‐CSF alone or in combination with KGF on oral epithelial differentiation of in vitro reconstituted normal human oral epithelium

**FIGURE 5:**
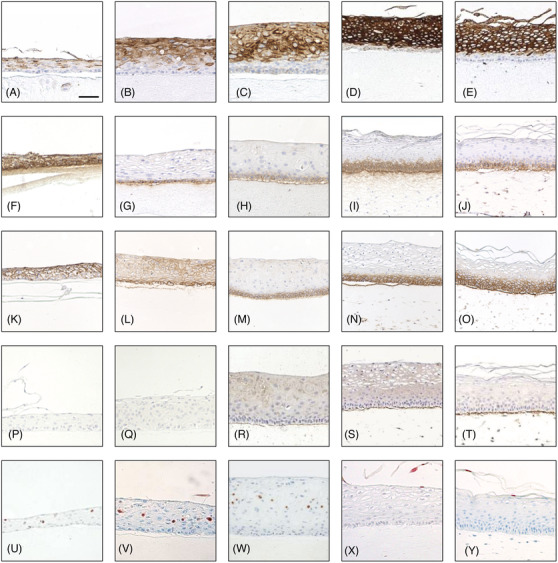
The effect of fibroblast‐derived diffusible factors (sandwich model) and granulocyte macrophage‐colony stimulating factor (GM‐CSF) alone or in combination with keratinocyte growth factor (KGF) on the phenotype of in vitro reconstituted normal human oral epithelium. The cultures were grown for 10 days in the absence (A, B, C, F, G, H, K, L, M, P, Q, R, U, V, X) or presence of fibroblasts in direct contact (E, J, O, T, Z) or at distance through a layer of simple collagen layer (sandwich models) – (D, I, N, S, Y) in the collagen matrix. Granulocyte macrophage‐colony stimulating factor (GM‐CSF) alone (B, G, L, Q, V) or in combination with keratinocyte growth factor (C, H, M, R, W) has been added to some of the three dimensional organotypic cultures in absence of fibroblasts. Immunohistochemistry for cytokeratin 13 (A‐E), β1‐integrin (F‐J), EGF receptor (K‐O), collagen IV (P‐T), and the TUNEL method (U‐Z) are shown. Scale bar = 50 µm

When grown in 3D monocultures on simple collagen gels, normal oral keratinocytes formed an epithelium with an undifferentiated phenotype (Figure 5, Table 2). Immunohistochemistry for various differentiation markers of these cultures revealed a weak, scattered expression of cytokeratin 13 (CK13, Figure 5A), strong expression of β1‐integrin (Figure 5F), and EGF receptor (EGF‐R, Figure 5K) throughout all cell layers with no deposition of collagen IV at the epithelium‐matrix interface (Figure 5P).

The presence of fibroblasts either in direct contact or at distance from the epithelium promoted formation of a fully maturated human buccal epithelium (Table 2) similar to the in vivo oral mucosa as judged after the panel of differentiation markers used in this study: uniform and strong expression of CK13 throughout all suprabasal epithelial cell layers (Figure 5D,E), polarization to the basal cell layer of β1 intergin (Figure 5I,J), and EGF‐R (Figure 5N,O), as well as synthesis and deposition of collagen IV at the epithelium‐matrix interface (Figure 5S,T). Addition of KGF (0.1‐100 ng/ml) did not change the undifferentiated phenotype of the oral epithelium grown on simple collagen gels, as previously reported by our group [1]. Addition of GM‐CSF (> 1 ng/ml) induced the expression of CK13 in all suprabasal cell layers (Figure 5B), and polarization of β1 integrin (Figure 5G) to the basal cell layer. The admixture of GM‐CSF and KGF (10 ng/ml each) induced, in addition, polarization of EGF‐R (Figure 5 M) to the basal cell layer and a fine deposition of collagen IV at the epithelium‐matrix interface (Figure 5R). This analysis shows that GM‐CSF alone or in combination with KGF was able to induce major aspects of oral epithelial differentiation of in vitro reconstituted normal human oral epithelium. None of the other growth factors tested in the study (EGF, TGFα, IL‐1α, HGF, SCF) did influence, when added, the phenotype of the epithelium grown on simple collagen gels.

### Effects of KGF and GM‐SCF on terminal differentiation of in vitro reconstituted normal human oral epithelium

The 3D monocultures of oral keratinocytes displayed TUNEL positive cells randomly distributed within the epithelium (Figure 5U). There was no polarization of TUNEL positive cells to the superficial layer, suggesting that cells did not complete the terminal stages of epithelial differentiation in these cultures. Similar pattern of distribution of TUNEL positive cells was also observed in the *3D* monocultures of oral keratinocytes supplemented with GM‐CSF alone or in combination with KGF (Figure 5V,X respectively). Polarization of TUNEL positive cells to the superficial cell layer was observed only when fibroblasts were present in the connective tissue equivalent, either in direct contact or at distance from the epithelium (Figure 5Y,Z). Addition of an anti‐GM‐CSF antibody to the culture medium of fibroblast‐containing cultures did not impair cell growth or the terminal differentiation of the reconstituted oral epithelium (Figure 2L). None of the other growth factors tested in the study (EGF, TGFα, IL‐1α, HGF, SCF) did influence, when added, the distribution of TUNEL positive cells within the epithelium grown on simple collagen gels. This analysis shows that terminal differentiation of in vitro reconstituted normal human oral epithelium was induced by underlying fibroblasts through diffusible factors, but not by the combination of KGF and GM‐CSF.

## DISCUSSION

Formation and maintenance of mature oral epithelium rely on a tightly balanced process of keratinocyte proliferation and terminal differentiation [14], but the knowledge about the specific factors involved is limited. Previously, we have developed a highly standardised serum free organotypic 3D‐OT model of human oral mucosa [15] and showed that fibroblasts are essential for differentiation of oral epithelium [1]. Data presented here further demonstrate that fibroblast‐derived diffusible factors are able to fully restore the differentiated phenotype of in vitro oral epithelium, including the fine‐tuned terminal stage of epithelial differentiation. From all the growth factors tested in the present study, alone or in various combinations, only GM‐CSF, alone or in combination with KGF, had a significant effect on the phenotype of oral epithelium. Previous reports from similar ‘3D organotypic’ models of skin morphogenesis and homeostasis [3, 16, 17] identified also GM‐CSF, alone or in combination with KGF, as a factor that induces a significant effect on the phenotype of epithelium. Of note, the skin 3D organotypic cultures supplemented with KGF only, displayed delays in expression of differentiation markers [18, 19]. Other reports showed that dermal keratinocytes treated with KGF exhibited increased proliferation as well, and inhibited differentiation, while reduced KGF levels restored the expression of differentiation markers [2]. One possible explanation is that the secretion of high doses of KGF by fibroblasts might influence the choice between proliferation and differentiation [19]. A limitation of the present study is that we did not test the effect of neutralizing antibodies for KGF, such that we could not infer more on the importance of this growth factor for the fine tuning of differentiation in oral epithelium.

The hematopoietic growth factor GM‐CSF is another growth factor that was found to regulate dermal keratinocyte growth and differentiation [8], playing an important role during the process of wound healing [20]. Fibroblast‐keratinocyte interactions in skin models strongly enhanced the expression of GM‐CSF [9], KGF and its receptor [21], while dermal keratinocyte‐released IL‐1 induced the expression of both KGF and GM‐CSF [10]. The regulatory mechanism of these two factors in skin homeostasis is a feedback loop within the multiple other epithelial‐mesenchymal interactions: skin keratinocytes release IL‐1α and IL‐1β, which stimulate the release of KGF and GM‐CSF by dermal fibroblasts. Then in turn, these two growth factors synthesized by dermal fibroblasts act on skin keratinocytes regulating their differentiation and proliferation [3, 7, 10].

In contrast to these reported observations from skin models proving that a combination if KGF and GM‐CSF can substitute for the dermal fibroblasts and provide sufficient support for both growth and differentiation of skin keratinocyte in their absence [3, 16, 17], our current study shows that the final stages of oral epithelial maturation could not be restored by KGF and GM‐CSF only. These differences between skin and oral mucosa morphogenesis might be due to the fact that oral mucosal fibroblasts and adult skin fibroblasts have different origin (the former originates from the neural crest and the latter from the mesoderm) and gene expressions, and consequently, different phenotypes and functions [22]. Oral fibroblasts were proven to express higher levels of KGF and to accelerate much faster the collagen gel contraction than dermal stromal cells [23, 24]. The results presented here, based on analysis of conditioned medium collected from oral fibroblasts maintained in *3D* monocellular cultures containing fibroblasts collagen matrices, show that oral fibroblasts synthesise considerable amounts of KGF, GM‐CSF, and HGF. These results corroborate with previous literature showing the oral fibroblasts to be the major producers of these growth factors [24, 25, 26], although oral keratinocytes have also been proven to synthesise GM‐CSF [27].

That the mesenchymal cell source has a significant influence on the thickness and ultrastructure of the epithelium has been previously shown [28]. Moreover, cytokeratin expression of the epithelial component was also proven to be strongly influenced by the origin of fibroblasts [29].

The data presented in this study indicate that in contrast to skin, other soluble factors than KGF and GM‐CSF released from fibroblasts exert the final tuning of oral epithelial differentiation. In support for this conclusion comes also the observation that addition of neutralizing antibodies against human GM‐CSF, previously shown to reduced keratinocyte proliferation and differentiation in skin models [7], did not impair cell proliferation or differentiation in our oral mucosa models. Taken together, the results of this study indicate that major aspects of oral epithelial differentiation are regulated by GM‐CSF in combination with KGF, but its terminal stage is controlled by another yet unidentified fibroblast‐derived diffusible factor.

## FUNDING

This work was funded by the Sectorial Operational Programme Human Resources Development (SOP HRD), financed from the European Social Fund, Romanian Government under the contract number SOP HRD/159/1.5/S/135760, Bergen Medical Research Foundation (Grant no. 20/2009), The Norwegian Cancer Research Association(Grant No. 515970/2011), Helse Vest (Grant No. 912260/2019), The Research Council of Norway through its Centers of Excellence funding scheme (Grant No. 22325), and The Norwegian Agency for International Cooperation and Quality Enhancement in Higher Education (project number CPEA‐LT‐2016/10106).

## AUTHOR CONTRIBUTIONS

**Conceptualization**: R Das, MJR Virlan, OK Vintermyr, AC Johannesen, B Calenic, DE Costea; **Investigation**: R Das, MJR Virlan, V Xenaki, KK Kulasekara, O Lukandu, E Neppelberg, OK Vintermyr; **Methodology**: R Das, KK Kulasekara, O Lukandu, DE Costea; **Data Curation**: R Das, MJR Virlan; **Formal Analysis**: R Das, MJR Virlan; **Funding Acquisition**: MJR Virlan, B Calenic, DE Costea; **Project Administration**: B Calenic, DE Costea; **Resources**: OK Vintermyr, AC Johannesen, B Calenic, DE Costea; **Software**: R Das, MJR Virlan; **Supervision**: OK Vintermyr, AC Johannesen, B Calenic, DE Costea; **Validation**: KK Kulasekara, O Lukandu, DE Costea; **Visualization**: R Das, MJR Virlan; **Writing ‐ Original Draft Preparation**: R Das, MJR Virlan; **Writing ‐ Review and Editing**: R Das, MJR Virlan, V Xenaki, KK Kulasekara, O Lukandu, E Neppelberg, OK Vintermyr, AC Johannesen, B Calenic, DE Costea.

## References

[1] Costea DE , Loro LL , Dimba EA , Vintermyr OK , Johannessen AC . Crucial effects of fibroblasts and keratinocyte growth factor on morphogenesis of reconstituted human oral epithelium. J Invest Dermatol. 2003;121:1479‐86. 14675199 10.1111/j.1523-1747.2003.12616.x

[2] Pickard A , Cichon AC , Menges C , Patel D , McCance DJ . Regulation of epithelial differentiation and proliferation by the stroma: a role for the retinoblastoma protein. J Invest Dermatol. 2012;132:2691‐9. 22696061 10.1038/jid.2012.201 PMC3443514

[3] Maas‐Szabowski N , Stark HJ , Fusenig NE . Keratinocyte growth regulation in defined organotypic cultures through IL‐1‐induced keratinocyte growth factor expression in resting fibroblasts. J Invest Dermatol. 2000;114:1075‐84. 10844548 10.1046/j.1523-1747.2000.00987.x

[4] Santosh AB , Jones TJ . The epithelial‐mesenchymal interactions: insights into physiological and pathological aspects of oral tissues. Oncol Rev. 2014;8:239. 10.4081/oncol.2014.239 25992230 PMC4419607

[5] Dongari‐Bagtzoglou A , Kashleva H . Development of a highly reproducible three‐dimensional organotypic model of the oral mucosa. Nat Protoc. 2006;1:2012‐8. 17487190 10.1038/nprot.2006.323 PMC2699620

[6] Calenic B , Ishkitiev N , Yaegaki K , Imai T , Costache M , Tovaru M , et al. Characterization of oral keratinocyte stem cells and prospects of its differentiation to oral epithelial equivalents. Rom J Morphol Embryol. 2010;51:641‐5. 21103620

[7] Maas‐Szabowski N , Shimotoyodome A , Fusenig NE . Keratinocyte growth regulation in fibroblast cocultures via a double paracrine mechanism. J Cell Sci. 1999;112:1843‐53. 10341204 10.1242/jcs.112.12.1843

[8] Maas‐Szabowski N , Szabowski A , Stark HJ , Andrecht S , Kolbus A , Schorpp‐Kistner M , et al. Organotypic cocultures with genetically modified mouse fibroblasts as a tool to dissect molecular mechanisms regulating keratinocyte growth and differentiation. J Invest Dermatol. 2001; 116:816‐20. 11348477 10.1046/j.1523-1747.2001.01349.x

[9] Zuliani T , Saiagh S , Knol AC , Esbelin J , Dreno B . Fetal fibroblasts and keratinocytes with immunosuppressive properties for allogeneic cell‐based wound therapy. PLoS One. 2013;8:e70408. 10.1371/journal.pone.0070408 23894651 PMC3722184

[10] Maas‐Szabowski N , Stärker A , Fusenig NE . Epidermal tissue regeneration and stromal interaction in HaCaT cells is initiated by TGF‐alpha. J Cell Sci. 2003;116:2937‐48. 12771184 10.1242/jcs.00474

[11] Kazimi BPS & Lewis MP Oral and dermal fibroblast organotypic co‐cultures show differences in phenotype. IADR 83rd General Session and Exhibition Baltimore, October 2005, Conference Abstract 0273. 2005.

[12] Loro LL , Vintermyr OK , Ibrahim SO , Idris AM , Johannessen AC . Apoptosis and expression of Bax and Bcl‐2 in snuff‐ and non‐snuff associated oral squamous cell carcinomas. Anticancer Res. 2000;20:2855‐60. 11062693

[13] Loro LL , Vintermyr OK , Johannessen AC . Cell death regulation in oral squamous cell carcinoma: methodological considerations and clinical significance. J Oral Pathol Med. 2003;32:125‐38. 12581382 10.1034/j.1600-0714.2003.00052.x

[14] Calenic B , Greabu M , Caruntu C , Tanase C , Battino M . Oral keratinocyte stem/progenitor cells: specific markers, molecular signaling pathways and potential uses. Periodontol 2000. 2015;69:68‐82. 26252402 10.1111/prd.12097

[15] Costea DE , Dimba AO , Loro LL , Vintermyr OK , Johannessen AC . The phenotype of in vitro reconstituted normal human oral epithelium is essentially determined by culture medium. J Oral Pathol Med. 2005;34:247‐52. 15752261 10.1111/j.1600-0714.2005.00308.x

[16] El Ghalbzouri A , Ponec M . Diffusible factors released by fibroblasts support epidermal morphogenesis and deposition of basement membrane components. Wound Repair Regen. 2004;12:359‐67. 15225215 10.1111/j.1067-1927.2004.012306.x

[17] Florin L , Knebel J , Zigrino P , Vonderstrass B , Mauch C , Schorpp‐Kistner M , et al. Delayed wound healing and epidermal hyperproliferation in mice lacking JunB in the skin. J Invest Dermatol. 2006;126:902‐11. 16439969 10.1038/sj.jid.5700123

[18] Andreadis ST , Hamoen KE , Yarmush ML , Morgan JR . Keratinocyte growth factor induces hyperproliferation and delays differentiation in a skin equivalent model system. FASEB J. 2001;15:898‐906. 11292649 10.1096/fj.00-0324com

[19] Lotti LV , Rotolo S , Francescangeli F , Frati L , Torrisi MR , Marchese C . AKT and MAPK signaling in KGF‐treated and UVB‐exposed human epidermal cells. J Cell Physiol. 2007;212:633‐42. 17458890 10.1002/jcp.21056

[20] Mann A , Niekisch K , Schirmacher P , Blessing M . Granulocyte‐macrophage colony‐stimulating factor is essential for normal wound healing. J Investig Dermatol Symp Proc. 2006;11:87‐92. 10.1038/sj.jidsymp.5650013 17069015

[21] Di CP , Sun Y , Zhao L , Li L , Ding C , Xu Y , et al. Effect of nifedipine on the expression of keratinocyte growth factor and its receptor in cocultured/monocultured fibroblasts and keratinocytes. J Periodontal Res. 2013;48:740‐7. 23528007 10.1111/jre.12064

[22] Mah W , Jiang G , Olver D , Cheung G , Kim B , Larjava H , et al. Human gingival fibroblasts display a non‐fibrotic phenotype distinct from skin fibroblasts in three‐dimensional cultures. PLoS One. 2014;9:e90715. 24608113 10.1371/journal.pone.0090715 PMC3946595

[23] Cichon AC , Pickard A , McDade SS , Sharpe DJ , Moran M , James JA , et al. AKT in stromal fibroblasts controls invasion of epithelial cells. Oncotarget. 2013;4:1103‐16. 23867201 10.18632/oncotarget.1078 PMC3759669

[24] Shannon DB , McKeown ST , Lundy FT , Irwin CR . Phenotypic differences between oral and skin fibroblasts in wound contraction and growth factor expression. Wound Repair Regen. 2006;14:172‐8. 16630106 10.1111/j.1743-6109.2006.00107.x

[25] Stephens P , Hiscox S , Cook H , Jiang WG , Zhiquiang W , Thomas DW . Phenotypic variation in the production of bioactive hepatocyte growth factor/scatter factor by oral mucosal and skin fibroblasts. Wound Repair Regen. 2001;9:34‐43. 11350638 10.1046/j.1524-475x.2001.00034.x

[26] Kim HS , Lee SG , Kim KH , Kim UK , Kim JR , Chung IK , et al. Production of GM‐CSF and TGF‐beta 1 in irradiated human gingival fibroblasts cultured with lipopolysaccharide. J Korean Assoc Oral Maxillofac Surg. 2002;28:169‐74.

[27] Gonzalez OA , Ebersole JL , Huang CB . Supernatants from oral epithelial cells and gingival fibroblasts modulate human immunodeficiency virus type 1 promoter activation induced by periodontopathogens in monocytes/macrophages. Mol Oral Microbiol. 2010;25:136‐49. 20331801 10.1111/j.2041-1014.2009.00552.x PMC4130208

[28] Kinikoglu B , Rovere MR , Haftek M , Hasirci V , Damour O . Influence of the mesenchymal cell source on oral epithelial development. J Tissue Eng Regen Med. 2012;6:245‐52. 21548135 10.1002/term.426

[29] Smola H , Stark HJ , Thiekotter G , Mirancea N , Krieg T , Fusenig NE . Dynamics of basement membrane formation by keratinocyte‐fibroblast interactions in organotypic skin culture. Exp Cell Res. 1998;239:399‐410. 9521858 10.1006/excr.1997.3910

